# Special Issue “Applied Sport Physiology and Performance—3rd Edition”

**DOI:** 10.3390/jfmk10010062

**Published:** 2025-02-13

**Authors:** W. Guy Hornsby

**Affiliations:** School of Sport Sciences, College of Applied Human Sciences, West Virginia University, Morgantown, WV 26505, USA; william.hornsby@mail.wvu.edu

## 1. Introduction and Overview

This Special Issue, “Applied Sport Physiology and Performance—3rd edition”, is a follow up to the previous two iterations (i.e., the 1st and 2nd editions). A major theme of these Special Issues has been studies involving competitive athletes, often captured in “real world” sporting environments, and reviews/education aimed at competitive sport. The present third edition of “Applied Sport Physiology and Performance” is no different. Overall, this Special Issue includes five original data articles, two of which were longitudinal, observational studies [1,2], two which were cross-sectional designs [3,4] and one that involved a controlled experiment [5]. The longitudinal studies included (1) female rugby athletes (international level) monitored (in game) across 3 years of competitions [1]; and (2) a case study on a competitive ultra-endurance athlete (male) [2]. The two cross-sectional studies included (1) elite judo athletes (male and female), and measures of various performance related variables [3]; and (2) assessment of shooting performance and performance-related kinematics in professional men’s basketball players [4]. Lastly, the lone experiment of this Special Issue involved the examination of caffeine’s effects on isokinetic strength, power, and endurance [5].

It is worth noting that several of these articles [1,2,3,4] included highly competitive and trained athletes (within the context of their sports). Long-term observational studies can afford coaches and sport scientists a better understanding of how athletes perform and respond to training, while cross-sectional assessment-based studies can help inform various aspects of performance and assessment/athlete monitoring.

In addition to the original data articles, this Special Issue includes three review articles on various aspects of training, assessment, and performance. Specifically, a scoping review on the isometric mid-thigh clean pull and the test’s relationship to dynamic performance [6]; a narrative review discussing training specificity and the strength-endurance continuum [7]; and a review of the lactate response to resistance training [8].

I applaud the authors for their efforts, and thank them for their contributions to this Special Issue.

## 2. A Brief Commentary on Sport Science

As this Special Issue series aims to aid, in a very small way, the field of sport science, with a focus on competitive sport, below is a brief commentary on exercise science vs. sport science, and how the “lens” of the sport scientist can influence their world view and beliefs.

## 3. A Comparison Between Exercise Science and Sport Science: A Personal Anecdote

One of my first jobs after completing my PhD was a faculty appointment at the Virginia Commonwealth University (VCU) within the Department of Kinesiology and Health Sciences (KHS), within their exercise science program. I arrived at VCU a year after spending 8 years (bachelors, masters, doctorate) at East Tennessee State University. Very generally, ETSU is a sport science program (sports performance-focused research, producing coaches and sport scientists, more aligned with the National Strength and Conditioning Association), and VCU is an exercise science program (which produces health/wellness/medical professionals, more aligned with the American College of Sports Medicine). And, at both VCU and ETSU, we had a weekly doctoral student seminar, in which research (published and potential future projects) would be presented and discussed by the doctoral students, department faculty, and sometimes outside researchers. Looking back, these were some of the most helpful, productive, and challenging scientific discussions I have been a part of, providing great lessons in critical thinking and humility. Observing the faculty critique each other’s work, often intensely (not in a mean-spirited manner) in an effort to improve a grant proposals or seeing doctoral students further into their studies than me possess high competency and spirited passion for their research topic(s) helped shaped me as a scientist. Perhaps most importantly, it showed me how fun and important science is.

When I began attending these meetings at VCU, I was struck by the contrast in the common themes of discussion compared to what I experienced at ETSU. At ETSU, the typical challenges of sport science research (e.g., needing trained subjects, working with athletic teams, challenges of not having a control and/or comparison group, small sample sizes, abnormal data, no money, etc.) were constantly a theme and, consequently, much of the conversation (and at times disagreement) centered around the associated challenges, such as statistics, interpretation/application, and hurdles for future studies. At VCU, in exercise science, the majority of the conversations centered around physiology and the related underlying mechanisms. I was amazed by how seemingly unconcerned they were, and how little time was spent discussing so many of the things that were constantly stressful and debated at ETSU. Reflecting on those early meetings at VCU, I was jealous.

## 4. The Influence of the Sport Scientists Lens

Sport performance is multifaceted, and is influenced by so many factors (to name a few: social, physiological, psychological, and training). Paraphrasing renowned sport scientist Iñigo Mujika, *if sport and sport science was simple, we could test athletes in a lab and then accurately predict the competition outcome. That we cannot is what makes sport and sport science so great* [9].

In sport science, most researchers likely have a background in sport to some degree (e.g., former competitive athlete). Beyond that, similar to how the world of sport is incredibly diverse (in terms of the number of different sports, the various cultures within sport, etc.), so too are the various backgrounds of those working in, teaching, and conducting research in sport science. There is no perfect starting point for becoming a sport scientist. We need diversity in thoughts, experiences, skill sets, and backgrounds. What is also needed is a passionate interest in sport, coupled with a high-level understanding/competency and an intense desire to substantially deepen knowledge. A sport scientist’s background and experience can influence how they view the world around them and, more specifically, how they view, interpret, and approach sport science research. Due to common challenges in sport science, touched on in the anecdote above and covered much better and in much more depth in many previous articles (see “Strength and Conditioning Journal Special Topic Issue on Sport Science and Performance”, *SCJ* volume 46, issue 1) [10], sport science often requires interpretation [11]. Thus, the background and resultant lens of the researcher can be highly influential. Often this is not well expressed when opposing viewpoints are discussed and the audience can easily miss key underlying reason(s) for conflicting ideas and stances. By nature, those working in sport tend to be competitive and passionate. Differing ideas between researchers are often perceived as a competition that can intensify into passionate argument. We must remind ourselves that—by definition—diversity of thought involves different opinions. Evaluating the concepts that form these opinions by applying the scientific process leads to an enormously positive effect upon the available knowledge that humans who study in sport possess. Theories are supported and refuted by the process of research as sufficient opportunities arise. Without healthy debates and collaboration, we all learn less.

We must all seek to retain our sense of citizenship in the scientific community, and embrace professional discourse. In the wise words of Mark Twain: “It is the mark of an educated mind to be able to entertain a thought without accepting it”. Perhaps it is also the mark of an educated mind to consider and test the concepts we may not agree with before we discount them, and tune our own lens to account for our own biases.
